# Nicotinamide Riboside Supplementation Alleviates Testicular Aging Induced by Disruption of Qprt‐Dependent NAD ^+^ De Novo Synthesis in Mice

**DOI:** 10.1111/acel.70004

**Published:** 2025-02-04

**Authors:** Yining Xu, Huan Wang, Hui Li, Chenlu Wei, Zhenye Zhu, Yanqing Zhao, Jiajia Zhu, Min Lei, Yingpu Sun, Qingling Yang

**Affiliations:** ^1^ Center for Reproductive Medicine The First Affiliated Hospital of Zhengzhou University Zhengzhou China; ^2^ Henan Key Laboratory of Reproduction and Genetics The First Affiliated Hospital of Zhengzhou University Zhengzhou China; ^3^ Henan Provincial Obstetrical and Gynecological Diseases (Reproductive Medicine) Clinical Research Center The First Affiliated Hospital of Zhengzhou University Zhengzhou China

**Keywords:** mitochondrial function, NAD^+^ de novo synthesis pathway, spermatogenesis, testicular aging

## Abstract

Recent studies have shown that disruptions in the nicotinamide adenine dinucleotide (NAD^+^) de novo synthesis pathway accelerate ovarian aging, yet its role in spermatogenesis remains largely unknown. In this study, we investigated the impact of the NAD^+^ de novo synthesis pathway on spermatogenesis by generating *Qprt*‐deficient mice using CRISPR‐Cas9 to target quinolinate phosphoribosyl transferase (*Qprt*), a key enzyme predominantly expressed in spermatocytes. Our results revealed that the deletion of *Qprt* did not affect NAD^+^ levels or spermatogenesis in the testes of 3‐month‐old mice. However, from 6 months of age onward, *Qprt*‐deficient mice exhibited significantly reduced NAD^+^ levels in the testes compared to wild‐type (WT) controls, along with a notable decrease in germ cell numbers and increased apoptosis. Additionally, these mice demonstrated mitochondrial dysfunction in spermatocytes, impaired progression through prophase I of meiosis, defective double‐strand break (DSB) repair, and abnormal meiotic sex chromosome inactivation. Importantly, supplementation with the NAD^+^ precursor nicotinamide riboside (NR) in *Qprt*‐deficient mice restored NAD^+^ levels and rescued the spermatogenic defects. These findings underscore the critical role of NAD^+^ de novo synthesis in maintaining NAD^+^ homeostasis and highlight its importance in meiotic recombination and meiotic sex chromosome inactivation in spermatogenesis.

## Graphical abstract

In *Qprt*‐deficient mice, testicular NAD^+^ levels decline accelerated with age, leading to mitochondrial dysfunction, disrupted DSB repair, impaired meiotic sex chromosome inactivation, and germ cell loss. Nicotinamide riboside (NR) supplementation restores NAD^+^ levels and alleviates these defects, highlighting the importance of NAD^+^ de novo synthesis in age‐related reproductive health.

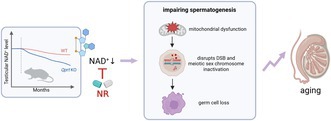

## 1 Introduction

Infertility is one of the global challenges today, with male factors accounting for approximately half of all infertility cases (Leifke and Nieschlag 1996; Marcho, Oluwayiose, and Pilsner 2020; Lin et al. 2024). Among these, infertility due to spermatogenic disorders affects about 9% of the male population worldwild (Boivin et al. 2007). Spermatogenesis involves three key processes, the self‐renewal and differentiation of spermatogonia, the meiosis of spermatocytes, and the maturation of spermatozoa (Thoma et al. 2013; Ramm and Schärer 2014). The meiosis of spermatocytes is crucial for the proper segregation of homologous chromosomes, and any abnormalities during this stage can result in meiotic arrest, impaired spermatogenesis, and ultimately male infertility (Practice Committee of the American Society for Reproductive Medicine 2015; Zhang et al. 2019b; Ishiguro 2024). Numerous studies have shown that defects in the formation and repair of DNA double‐strand breaks (DSBs) in spermatocytes can lead to impairments of meiotic recombination, increased apoptosis and reduced fertility (Oakberg 1971; Lanfranco et al. 2004; Ge, Chen, and Hardy 2008; Griswold 2016; Soh et al. 2017). During spermatogenesis, the transition from mitosis to meiosis requires significant metabolic reprogramming, including shifts in energy substrate utilization and changes in mitochondrial dynamics (Varuzhanyan and Chan 2020). This regulation is influenced by various signaling pathways that respond to intrinsic and extrinsic cues, ensuring that spermatocytes meet the energetic demands of meiosis (Vertika, Singh, and Rajender 2020). Additionally, factors such as reactive oxygen species (ROS) production and the balance between anabolism and catabolism within mitochondria are vital for maintaining cellular homeostasis during this process (Shadel and Horvath 2015; Guo et al. 2022; Zhu et al. 2023). Understanding how mitochondrial metabolism regulates spermatocyte meiosis offers insights into male fertility and highlights potential therapeutic targets for reproductive disorders.

Nicotinamide adenine dinucleotide (NAD^+^) is a vital coenzyme involved in numerous energy metabolism pathways, including glycolysis, the tricarboxylic acid cycle, and fatty acid oxidation (Tegelenbosch and de Rooij 1993; Xiao et al. 2018; Covarrubias et al. 2021). Recent studies have demonstrated that decreased NAD^+^ levels can impair mitochondrial function, leading to DNA damage and the release of ROS (Inoue et al. 2014; Huang et al. 2020). Furthermore, reduced NAD^+^ concentrations can diminish the activity of DNA repair enzymes, thereby exacerbating genomic instability (Ruszkiewicz, Bürkle, and Mangerich 2022). In mammals, NAD^+^ synthesis occurs primarily through three pathways, the Preiss‐Handler pathway, the kynurenine pathway (de novo pathway), and the salvage pathway (Zickler and Kleckner 2015; Amjad et al. 2021). Relevant studies have indicated that NAD^+^ de novo synthesis enhances mitochondrial function (Katsyuba et al. 2018). Genetic or pharmacological blockade of this pathway in macrophages results in decreased intracellular NAD^+^ levels, negatively impacting NAD^+^‐dependent signaling and respiratory functions in mitochondria. This impairment ultimately compromises the phagocytic and anti‐inflammatory capabilities of macrophages (Griswold 2016). Our previous study found that disruption of the NAD^+^ de novo synthesis pathway accelerates ovarian aging in mice (Yang et al. 2023); however, the role of this pathway in testicular spermatogenesis, particularly during meiosis in spermatocytes, remains unclear.

Here, we found that *Qprt*, the key enzyme in the NAD^+^ de novo synthesis pathway, is highly expressed in spermatocytes. Utilizing CRISPR‐Cas9 technology, we generated *Qprt* knockout (*Qprt* ^−/−^) mice. At 3 months of age, testicular NAD^+^ levels in these mice were nearly normal. However, by 6 months, the absence of *Qprt* led to a significant decrease in NAD^+^ levels, resulting in increased apoptosis. Mechanistically, *Qprt* knockout mice exhibited mitochondrial dysfunction in spermatocytes, impaired progression through prophase I of meiosis, defective DSB repair, and abnormal meiotic sex chromosome inactivation. Notably, supplementation with NR in *Qprt* knockout mice restored NAD^+^ levels and reversed spermatogenic defects. These findings suggest the critical role of the NAD^+^ de novo synthesis pathway in maintaining NAD^+^ homeostasis and highlight the importance of NAD^+^ in regulating meiotic recombination and meiotic sex chromosome inactivation during spermatogenesis.

## 2 Results

### 2.1 *Qprt* Deletion Accelerated Testicular Aging in Mice

**FIGURE 1:**
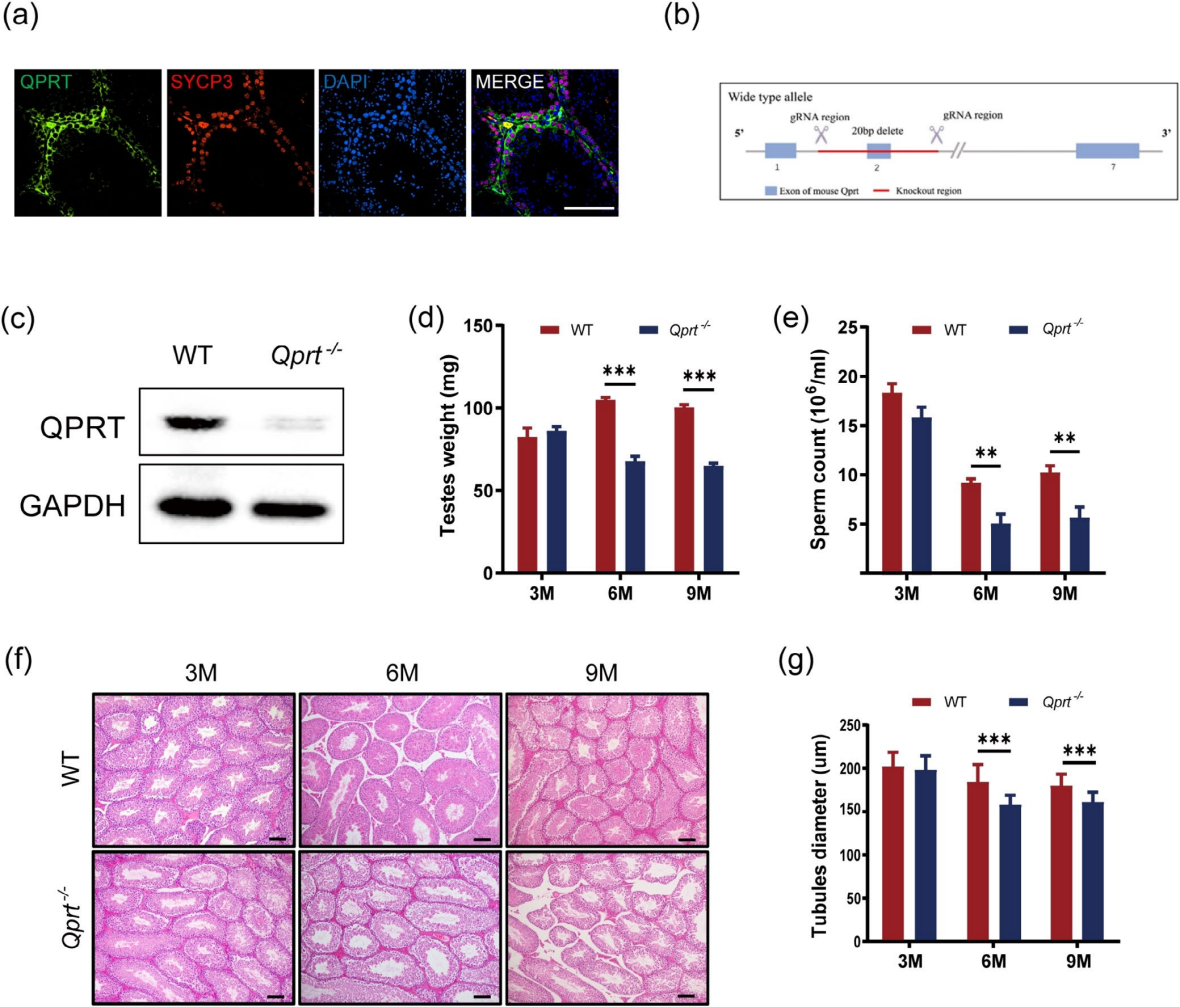
*Qprt* deletion accelerated testicular aging. (a) Immunofluorescence staining of testis sections shows the localization of QPRT (green), SYCP3 (red) and DAPI (blue). Scale bar = 100 μm. (b) Schematic representation of the *Qprt* knockout allele generated using CRISPR‐Cas9 technology. (c) Western blot analysis confirms the absence of QPRT protein in the testes of *Qprt* ^−/−^ mice compared to WT controls, with GAPDH serving as a loading control. (d) Testis weights at 3, 6, and 9 months of age from WT and *Qprt* ^−/−^ mice (*n* = 8 mice for each group). (e) Sperm counts from WT and *Qprt* ^−/−^ mice at 3, 6, and 9 months of age (*n* = 6 mice for each group). (f) Hematoxylin and eosin (H&E) staining of testicular sections from WT and *Qprt* ^−/−^ mice at 3, 6, and 9 months of age. Scale bar = 100 μm. (g) Quantification of seminiferous tubule diameters in WT and *Qprt* ^−/−^ mice at 3, 6, and 9 months of age (*n* = 61 convoluted seminiferous tubules from 3 mice per group). *p < 0.05, **p < 0.01, ***p < 0.001. Data are presented as means ± SEM.

We initially assessed the expression of *Qprt* within seminiferous tubules using immunofluorescence staining. We found that *Qprt* is predominantly localized in spermatocytes, as identified by SYCP3 staining (Figure 1a), suggesting a potential role for this protein in spermatogenesis. To further validate this hypothesis, we utilized CRISPR‐Cas9 technology to generate a global *Qprt* knockout mouse model (Figure 1b). The absence of QPRT protein expression was confirmed through the isolation and analysis of testis from the knockout mice (Figure 1c). We subsequently monitored testis weight, sperm count, and sperm morphology in Qprt^−/−^ and WT mice. Compared to controls, both testis weight and sperm count were significantly reduced in 6‐ and 9‐month‐old *Qprt* ^−/−^ mice, while no notable differences were observed at 3 months of age (Figure 1d,e). However, the analysis of sperm morphology in Qprt^−/−^ and WT mice at 9 months of age revealed no significant differences between the two groups (Figure S1). Additionally, at 3 months, the seminiferous tubule diameter in *Qprt* ^−/−^ mice was comparable to that of age‐matched controls. However, after 6 months, the tubule diameter in *Qprt* ^−/−^ mice showed a significant reduction (Figure 1f,g). Furthermore, considering that Leydig cell functions are decreased during aging and are likely affected by abnormalities in germ cells in the *Qprt* ^−/−^ mice, we measured the serum testosterone levels in *Qprt* ^−/−^ and WT mice at 9 months of age, but found no significant differences between the two groups (Figure S2), indicating that *Qprt* knockout does not significantly affect Leydig cell function. These findings highlight the crucial role of *Qprt* in maintaining testicular function, with its deletion accelerating testicular aging in mice.

### 2.2 *Qprt* Deletion Led to Germ Cell Loss in Mice

**FIGURE 2:**
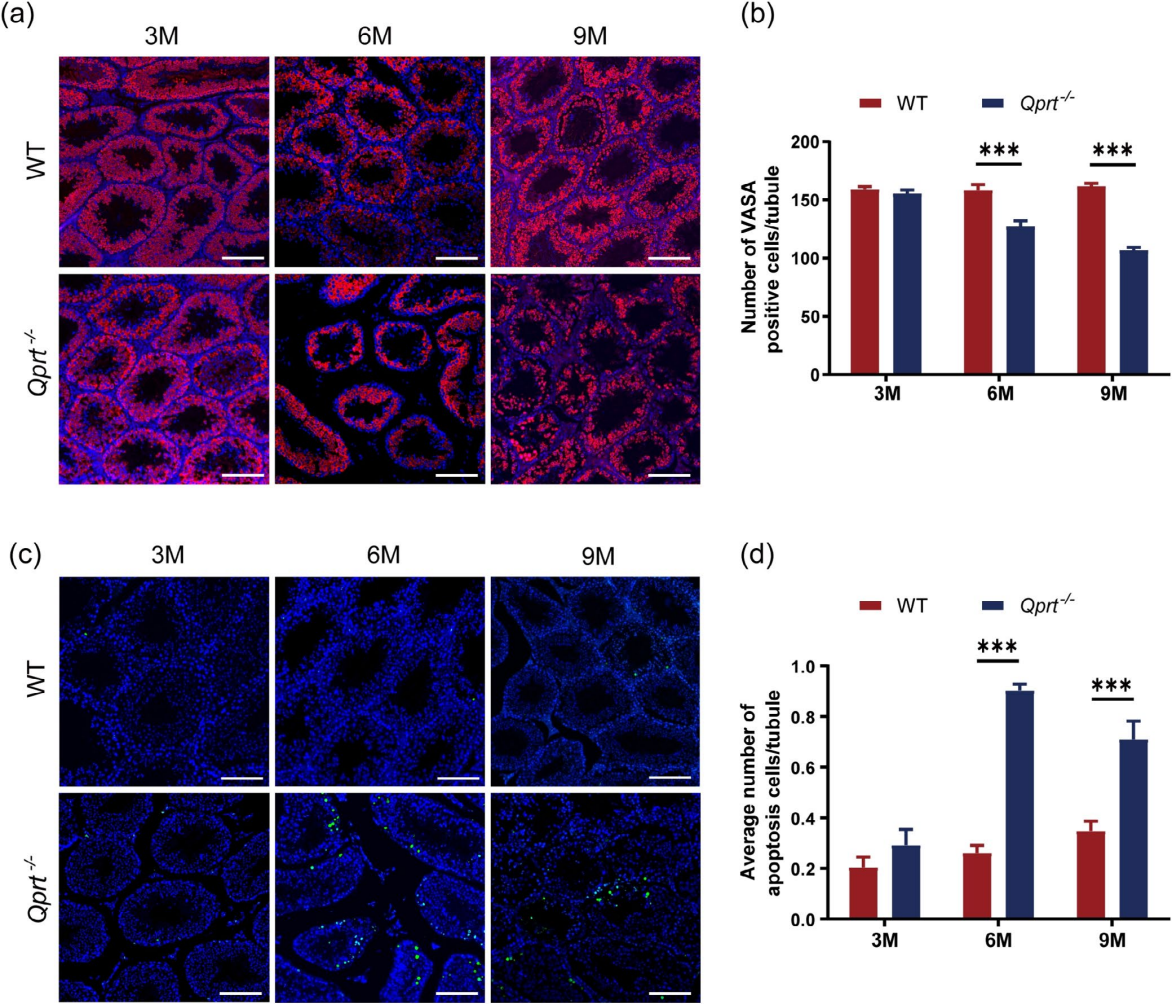
*Qprt* deletion led to a reduction in testicular germ cell numbers with aging in mouse testis. (a) Representative immunofluorescence staining for VASA (red) with DAPI (blue) counterstaining in the testes of WT and *Qprt* ^−/−^ mice at 3, 6, and 9 months of age. Scale bar = 100 μm. (b) Quantification of VASA‐positive cells per tubule at 3, 6, and 9 months of age (*n* = 61 convoluted seminiferous tubules from 3 mice per group). (c) Representative TUNEL staining (green) with DAPI counterstaining (blue) in testis sections from WT and *Qprt* ^−/−^ mice at 3, 6, and 9 months of age. Scale bar = 100 μm. (d) The average number of apoptotic cells per seminiferous tubule at 3, 6, and 9 months (*n* = 3–4 mice per group; at least 50 seminiferous tubules were counted per mouse in each group). *p < 0.05, **p < 0.01, ***p < 0.001. Data are presented as means ± SEM.

To further confirm that *Qprt* deletion accelerates testicular aging, we performed immunofluorescence staining using the germ cell marker VASA on testicular sections from WT and *Qprt* ^−/−^ mice at 3, 6, and 9 months of age (Figure 2a). As shown in Figure 2b, the number of germ cells in the seminiferous tubules of *Qprt* ^−/−^ mice was significantly reduced at 6 and 9 months compared to controls, while no such reduction was observed in 3‐month‐old mice. Additionally, cell apoptosis was assessed by TUNEL staining on testicular sections (Figure 2c). There was no significant difference in the number of apoptotic cells per seminiferous tubule between 3‐month‐old *Qprt* ^−/−^ and WT mice, but a marked increase in apoptotic cells, predominantly spermatocytes, was observed at 6 and 9 months in *Qprt* ^−/−^ mice (Figure 2d). These results further support that *Qprt* deletion accelerates testicular aging by impairing germ cell survival.

### 2.3 Deletion of *Qprt* Led to Mitochondrial Dysfunction in Spermatocytes

**FIGURE 3:**
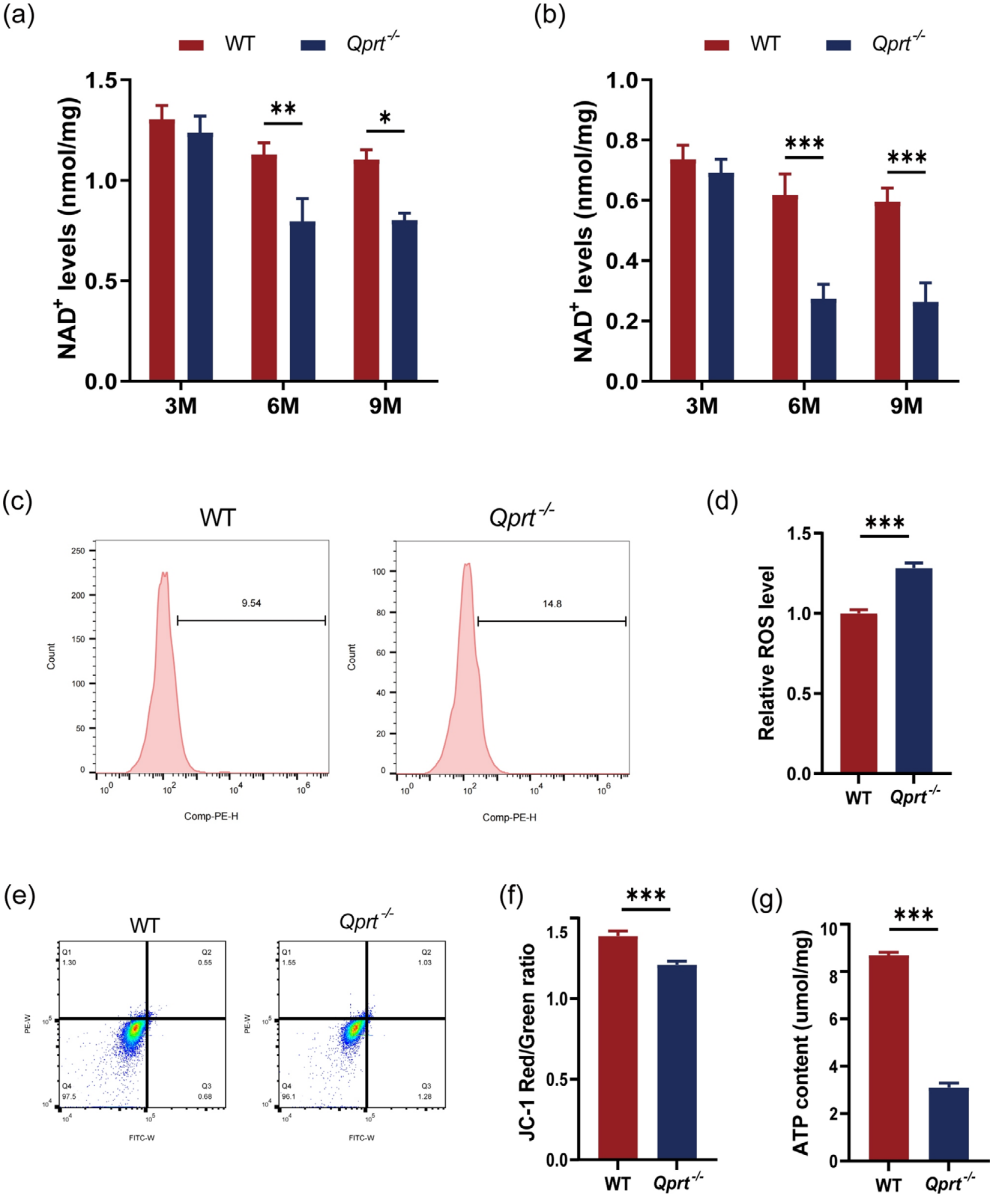
*Qprt* deletion led to mitochondrial dysfunction in spermatocytes. (a) NAD^+^ levels in testes from WT and *Qprt* ^−/−^ mice at 3, 6, and 9 months of age (*n* = 6 mice per group). (b) NAD^+^ levels in pachytene spermatocytes from WT and *Qprt* ^−/−^ mice at 3, 6, 9 months of age (*n* = 6 mice for each group). (c) Representative flow cytometry plots showing reactive oxygen species (ROS) levels in pachytene spermatocytes from WT and *Qprt* ^−/−^ mice at 9 months of age. (d) Relative ROS levels in two groups at 9 months (*n* = 6 mice for each group). (e) JC‐1 staining flow cytometry shows mitochondrial membrane potential in pachytene spermatocytes from WT and *Qprt* ^−/−^ groups at 9 months. (f) Quantification of the JC‐1 Red/Green fluorescence ratio representing mitochondrial membrane potential in different groups at 9 months of age (*n* = 6 mice per group). (g) ATP content measurement in pachytene spermatocytes from WT and *Qprt* ^−/−^ mice at 9 months old age (*n* = 6 mice per group). *p < 0.05, **p < 0.01, ***p < 0.001. Data are presented as means ± SEM.

We next measured NAD^+^ levels in 3, 6, and 9‐month‐old mice (Figure 3a,b). Compared to WT mice, we found that *Qprt* deletion did not significantly affect NAD^+^ levels in the testes or pachytene spermatocytes of 3‐month‐old mice. However, at 6 and 9 months of age, NAD^+^ levels were significantly reduced in *Qprt* knockout mice. These results suggest that disrupting NAD^+^ de novo synthesis by deleting *Qprt* does not significantly impact early testicular function. Based on these findings, we proceeded with further experiments using mice older than 6 months.

To investigate whether abnormalities in spermatogenesis are caused by reduced NAD^+^ levels leading to mitochondrial dysfunction in germ cells, we isolated pachytene spermatocytes from the testes of 9‐month‐old mice and analyzed them using flow cytometry. The results showed an increased reactive oxygen species (ROS) content in pachytene spermatocytes of *Qprt* knockout mice compared to the WT controls (Figure 3c,d). Additionally, we assessed mitochondrial membrane potential and ATP levels in pachytene spermatocytes from 9‐month‐old mice. As shown in Figure 3e,f, *Qprt* knockout mice exhibited a lower ratio of red to green fluorescence, indicating a reduced mitochondrial membrane potential, along with significantly decreased ATP levels (Figure 3g). These findings suggest that decreased NAD^+^ levels impair mitochondrial function in germ cells, resulting in mitochondrial metabolic abnormalities.

### 2.4 Deletion of *Qprt* Led to Abnormal Meiotic Recombination and Meiotic Sex Chromosome Inactivation in Spermatocytes

**FIGURE 4:**
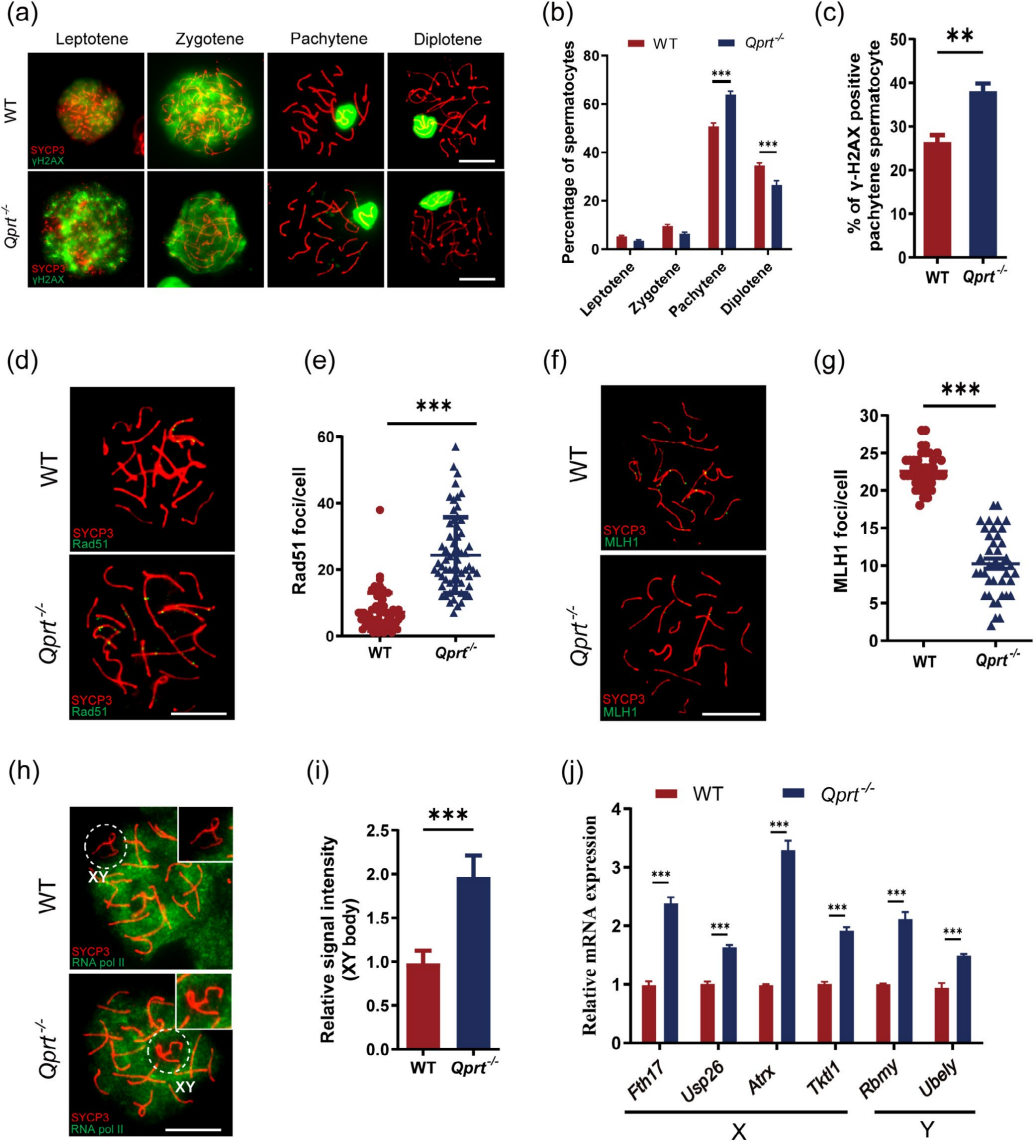
*Qprt* deletion disrupted meiotic recombination and meiotic sex chromosome inactivation in spermatocytes. (a) Representative images of spermatocytes at sub stages of meiotic prophase I in WT and *Qprt* ^−/−^ mice, including leptotene, zygotene, pachytene, and diplotene. SYCP3 (red) marks the chromosome axes, and γH2AX (green) indicates DSBs. Scale bar = 10 μm. (b) Quantification of the percentage of spermatocytes at different meiotic stages in WT and *Qprt* ^−/−^ mice at 6 months of age (*n* = 5 mice for each group, at least 300 spermatocytes counted per mouse). (c) Percentage of γH2AX‐positive on autosomes in pachytene spermatocytes in both groups (*n* = 5 mice for each group, at least 80 pachytene spermatocytes were counted per mouse). (d) Immunofluorescence staining of SYCP3 (red) and Rad51 (green) in pachytene spermatocytes. Scale bar = 10 μm. (e) Quantification of Rad51 foci per cell in the 6‐month‐old WT and *Qprt* ^−/−^mice (*n* = 63 pachytene spermatocytes were counted per group). (f) Immunofluorescence staining of SYCP3 (red) and MLH1 foci (green) in pachytene spermatocytes. Scale bar = 10 μm. (g) Quantification of MLH1 foci per cell in 6‐month‐old WT and *Qprt* ^−/−^ mice (*n* = 40–44 pachytene spermatocytes were counted per group). (h) RNA polymerase II (RNA pol II) staining (green) in the XY body and SYCP3 staining (red) of pachytene spermatocytes. The XY body is highlighted in the inset. Scale bar = 10 μm. (i) Relative average RNA pol II signal intensity in the XY body of pachytene spermatocytes between 6‐month‐old WT and *Qprt* ^−/−^ mice (*n* = 60 pachytene spermatocytes were counted per group). (j) Relative mRNA expression levels of X‐ and Y‐linked genes in pachytene spermatocytes from 6‐month‐old WT and *Qprt* ^−/−^ mice, as determined by qRT‐PCR (*n* = 6 mice for each group). *p < 0.05, **p < 0.01, ***p < 0.001. Data are presented as means ± SEM.

To investigate whether the decrease in NAD^+^ levels mediated by *Qprt* deletion affects meiotic recombination in spermatocytes, we first assessed the progression of prophase I in these cells. We prepared chromosome spreads of spermatocytes and performed immunofluorescence staining using the synaptonemal complex protein SYCP3 (red) and the DNA double‐strand break (DSB) marker γH2AX (green). Statistical analysis showed that, compared to WT mice, *Qprt* ^−/−^ mice exhibited an increased proportion of spermatocytes in the pachytene stage and a significant decrease in the proportion of cells in the diplotene stage (Figure 4a,b). Additionally, the proportion of pachytene spermatocytes with persistent γH2AX staining on autosomes was significantly higher in *Qprt* ^−/−^ mice (Figure 4a,c). To further confirm that *Qprt* gene knockout impairs meiotic recombination, we conducted immunofluorescence staining on spermatocyte spreads using SYCP3 (red) and the DSB homologous repair‐related enzyme Rad51 (green). The results demonstrated a significant increase in the number of Rad51 foci on autosomes of pachytene spermatocyte form *Qprt* ^−/−^ mice compared to age‐matched WT mice (Figure 4d,e). MLH1 is a crucial marker for meiotic recombination, indicating crossover events (Baker et al. 1996; Guillon et al. 2005; Hassold et al. 2009). Immunofluorescence staining of spermatocyte spreads with SYCP3 and MLH1 revealed a marked reduction in MLH1 foci on the chromosomes of pachytene spermatocytes in *Qprt* ^−/−^ mice compared to WT controls (Figure 4f,g). These findings indicate that *Qprt* deletion disrupted the progression of prophase I, leading to abnormalities in meiotic recombination of spermatocytes.

Meiotic sex chromosome inactivation is crucial for maintaining genomic stability during spermatogenesis, as it silences unsynapsed sex chromosomes, preventing the expression of potentially harmful genes and ensuring proper meiotic progression (Richler et al. 1994; Khalil, Boyar, and Driscoll 2004). To further confirm whether the decrease in NAD^+^ levels induced by *Qprt* deletion affects meiotic sex chromosome inactivation, we performed immunofluorescence staining on spread spermatocytes using SYCP3 (red) and RNA polymerase II (green). The results showed that, compared to WT mice, the distribution of RNA polymerase II in the sex chromosome regions (XY body) was markedly increased in *Qprt* knockout mice (Figure 4h,i). To investigate whether this abnormal RNA polymerase II localization correlates with changes in gene expression, we performed qRT‐PCR analysis of X‐ and Y‐chromosome‐related genes in pachytene spermatocytes. The results revealed that expression of all the genes tested, including *Fth17, Usp26*, *Atrx*, *Tktl1* and *Rbmy*, *Ubely* was significantly upregulated in *Qprt* knockout mice compared to controls (Figure 4j). This suggests that *Qprt* deletion disrupted the proper silencing of sex chromosome genes during meiotic sex chromosome inactivation. Collectively, these findings indicate that *Qprt* knockout leads to abnormal meiotic sex chromosome inactivation in spermatocytes.

### 2.5 Supplementation With NR Increased NAD ^+^ Levels and Restored Spermatogenesis in *Qprt* ^−/−^ Male Mice

**FIGURE 5:**
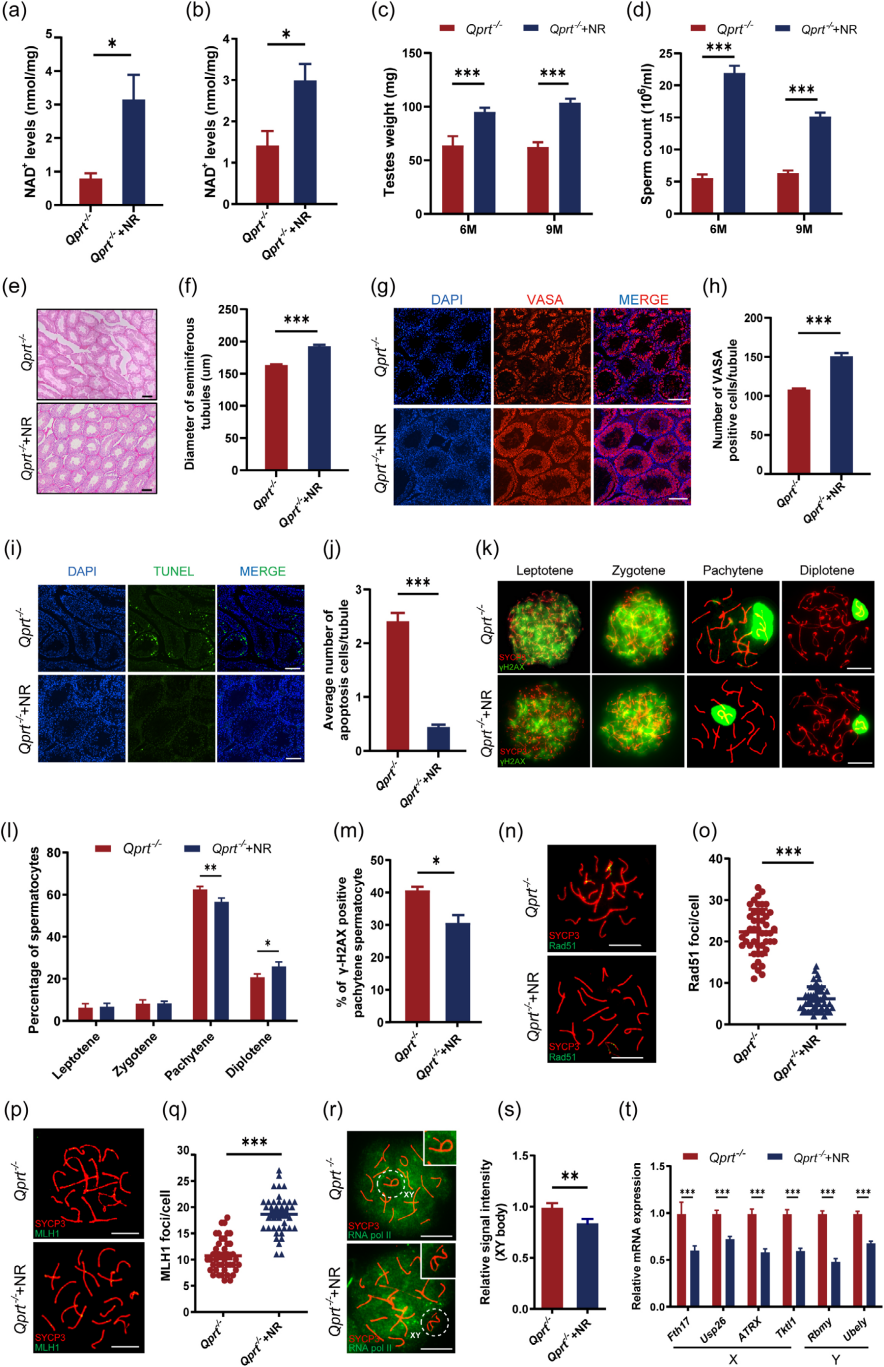
NR supplementation increased testicular NAD^+^ levels and decreased spermatogenesis defects in *Qprt* ^−/−^ mice. (a, b) NAD^+^ content in pachytene spermatocytes (a) and testis (b) from 6‐month‐old *Qprt* ^−/−^ and NR‐supplemented *Qprt* ^−/−^ mice. (*n* = 3 mice per group). (c) Testis weights in 6‐ and 9‐month‐old *Qprt* ^−/−^ and NR‐supplemented *Qprt* ^−/−^ mice (*n* = 6 mice per group). (d) Sperm count in 6‐ and 9‐month‐old *Qprt* ^−/−^ and NR‐supplemented *Qprt* ^−/−^ mice (*n* = 6 mice per group). (e) Representative hematoxylin and eosin (H&E) staining of testicular sections from 9‐month‐old *Qprt* ^−/−^ and NR‐supplemented *Qprt* ^−/−^ mice, showing seminiferous tubule structure. Scale bar = 100 μm. (f) Measurement of seminiferous tubule diameter in *Qprt* ^−/−^ and NR‐supplemented *Qprt* ^−/−^ mice at 9 months (*n* = 65 seminiferous tubules from 3 mice per group). (g) Representative immunofluorescence images of testicular sections from 9‐month‐old *Qprt* ^−/−^ and NR‐supplemented *Qprt* ^−/−^ mice stained for VASA (red) and DAPI (blue). Scale bar = 100 μm. (h) Quantification of VASA‐positive cells per seminiferous tubule in the testes of 9‐month‐old *Qprt* ^−/−^ and NR‐supplemented *Qprt* ^−/−^ mice (*n* = 65 seminiferous tubules from 3 mice per group). (i) TUNEL staining (green) with DAPI counterstaining (blue) in testicular sections from 9‐month‐old *Qprt* ^−/−^ and NR‐supplemented *Qprt* ^−/−^ mice. Scale bar = 100 μm. (j) Quantification of apoptotic cells per seminiferous tubule in 9‐month‐old *Qprt* ^−/−^ and NR‐supplemented *Qprt* ^−/−^ mice (*n* = 3 mice per group). (k) Immunofluorescence staining of SYCP3 (red) and γH2AX (green) in spermatocytes at different stages of prophase I from *Qprt* ^−/−^ and NR‐supplemented *Qprt* ^−/−^ mice. Scale bar = 10 μm. (l) Percentage of spermatocytes at different prophase I stages in 9‐month‐old *Qprt* ^−/−^ and NR‐supplemented *Qprt* ^−/−^ mice (*n* = 3 mice per group). (m) Percentage of γH2AX‐positive pachytene spermatocytes in 9‐month‐old *Qprt* ^−/−^ and NR‐supplemented *Qprt* ^−/−^ mice (*n* = 3 mice per group). (n, o) Immunofluorescence staining of SYCP3 (red) and Rad51 (green) in pachytene spermatocytes from 9‐month‐old *Qprt* ^−/−^ and NR‐supplemented *Qprt* ^−/−^ mice, and quantification of Rad51 foci per cell (*n* = 46–50 pachytene spermatocytes per group). Scale bar = 10 μm. (p, q) Representative images of immunofluorescence staining of SYCP3 (red) and MLH1 (green) in pachytene spermatocytes from 9‐month‐old *Qprt* ^−/−^ and NR‐supplemented *Qprt* ^−/−^ mice, and quantification of MLH1 foci per cell (*n* = 41–42 pachytene spermatocytes per group). Scale bar = 10 μm. (r, s) Representative images of immunofluorescence staining of SYCP3 (red) and RNA pol II (green) in pachytene spermatocytes from 9‐month‐old *Qprt* ^−/−^ and NR‐supplemented *Qprt* ^−/−^ mice, with insets showing magnified XY body regions. Relative RNA pol II signal intensity in the XY body (*n* = 67 pachytene spermatocytes per group). Scale bar = 10 μm. (t) Relative mRNA expression levels of X‐ and Y‐linked genes in 9‐month‐old *Qprt* ^−/−^ and NR‐supplemented *Qprt* ^−/−^ spermatocytes, as determined by qRT‐PCR (*n* = 9 mice per group). *p < 0.05, **p < 0.01, ***p < 0.001. Data are presented as means ± SEM.

Nicotinamide Riboside (NR) is an important precursor in the NAD^+^ biosynthesis pathway, generating NAD^+^ through the salvage pathway. The effects of NR on various systems in the body have been well‐documented in previous studies (Yoshino, Baur, and Imai 2018). To further confirm the impact of NAD^+^ on spermatogenesis, we supplemented *Qprt* ^−/−^ mice with NR starting at 4 weeks of age and continued for 5–8 months. As shown in Figure 5a,b, NR supplementation significantly increased NAD^+^ levels in both isolated pachytene spermatocytes and testes of 6‐month‐old *Qprt* ^−/−^ mice compared to age‐matched controls. Additionally, testicular weight and sperm count were significantly elevated in NR‐treated mice (Figure 5c,d). Hematoxylin and eosin (H&E) staining revealed that NR supplementation significantly increased the diameter of seminiferous tubules (Figure 5e,f) and the number of germ cells, as identified by VASA staining (Figure 5g,h). Consequently, the number of apoptotic cells was notably reduced in the *Qprt* ^−/−^ mice following NR supplementation (Figure 5i,j). These results indicate that NR supplementation partially reverses spermatogenic defects observed in *Qprt* knockout mice.

To further assess the impact of NR supplementation on meiotic progression and recombination, we analyzed spermatocytes from both *Qprt* ^−/−^ mice and NR‐supplemented *Qprt* ^−/−^ mice. Spermatocytes were spread and subjected to immunofluorescence staining. The γH2AX staining revealed that, compared to age‐matched *Qprt* ^−/−^ mice, which exhibited a higher proportion of pachytene spermatocytes, NR supplementation restored the proportion of pachytene spermatocytes, reduced the percentage of pachytene spermatocytes with positive γH2AX staining on autosomes, and increased the proportion of diplotene spermatocytes (Figure 5k–m). These findings suggest that NR supplementation partially restores meiotic prophase I progression disrupted by *Qprt* knockout. Furthermore, Rad51 and MLH1 immunofluorescence staining indicated that NR supplementation significantly reduced abnormalities in chromosomal double‐strand break (DSB) repair and crossover formation in spermatocytes from *Qprt* knockout mice (Figure 5n–q). Regarding sex chromosome gene silencing, NR‐supplemented *Qprt* ^−/−^ mice exhibited a marked reduction in RNA polymerase II staining intensity in the sex body regions compared to knockout mice (Figure 5r,s). Similarly, the expression levels of X‐ and Y‐chromosome‐related genes were significantly reduced (Figure 5t). These results demonstrate that NR supplementation ameliorates meiotic prophase I abnormalities and restores normal sex chromosome gene silencing in spermatocytes from *Qprt* ^−/−^ mice.

## 3 Discussion

In this study, we have demonstrated the crucial role of NAD^+^ homeostasis, particularly through the de novo synthesis pathway mediated by *Qprt*, in maintaining spermatogenesis with age. The deletion of *Qprt* led to progressive declines in NAD^+^ levels, particularly after 6 months of age, which were associated with significant defects in germ cell survival and mitochondrial function in spermatocytes. These disruptions manifested as impaired progression through meiosis, defective DNA double‐strand break repair, and abnormal meiotic sex chromosome inactivation. Our findings also highlight the therapeutic potential of NAD^+^ precursor supplementation, as nicotinamide riboside effectively rescued the observed spermatogenic abnormalities in *Qprt*‐deficient mice, emphasizing the importance of NAD^+^ in reproductive health and aging.

NAD^+^ can be synthesized through three pathways: the Preiss‐Handler pathway, the salvage pathway, and the de novo pathway (Liu et al. 2018; Harjes 2019). In the de novo pathway, the essential amino acid tryptophan serves as a substrate, with *Qprt* catalyzing the formation of nicotinic acid mononucleotide, which is subsequently converted into NAD^+^ via a series of enzymatic reactions in the Preiss‐Handler pathway. Coordinated regulation of these three pathways is crucial for maintaining intracellular NAD^+^ levels, which are essential for cellular function, a decline in NAD^+^ levels can lead to various pathological and physiological conditions (Minhas et al. 2019; Zhang et al. 2019a). In this study, we identified that *Qprt*, the rate‐limiting enzyme in the NAD^+^ de novo synthesis pathway, is predominantly expressed in spermatocytes within the testes. *Qprt* knockout resulted in reduced testis weight and decreased sperm count in 6‐ and 9‐month‐old mice, while no such effects were observed in 3‐month‐old mice. NAD^+^ level measurements showed no significant reduction in the testes or spermatocytes of 3‐month‐old *Qprt*‐deficient mice. However, a substantial decline was detected by 6 months of age. These findings suggest that during early testicular development, the NAD^+^ de novo synthesis pathway is not critical for maintaining NAD^+^ levels in the testes. This observation aligns with our previous findings in ovaries, where NAD^+^ levels remained unchanged at 3 months of age despite disruption of the de novo synthesis pathway (Yang et al. 2023). Additionally, a study using the acquired niacin dependency (ANDY) model, which experimentally lowers NAD^+^ levels. In this model, reducing NAD^+^ levels in young adult mice to levels resembling the natural decline seen in aging mice disrupts spermatogenesis, leading to smaller testes and decreased sperm counts(Meyer‐Ficca et al. 2022). Collectively, these results highlight that the NAD^+^ de novo synthesis pathway becomes essential for maintaining NAD^+^ levels and reproductive function in both the testes and ovaries as organisms age.

Mitochondria are known as the energy factories of cells (Labarta et al. 2019; Xia et al. 2019) and are closely linked to sperm function and male fertility (Evenson, Darzynkiewicz, and Melamed 1982; Agnihotri et al. 2016; Vahedi Raad et al. 2024). NAD^+^ plays a crucial role as a coenzyme in redox reactions of the tricarboxylic acid cycle, driving oxidative phosphorylation for ATP production and regulating intracellular ROS balance (Lee et al. 2019; Myakala et al. 2023; Waddell, Khatoon, and Kristian 2023). Given the essential role of NAD^+^ in mitochondrial function, as evidenced by the fact that NAD^+^ deficiency leads to mitochondrial dysfunction (Wiley et al. 2016; Liu et al. 2021; Wang et al. 2021), we also analyzed mitochondrial activity in *Qprt* ^−/−^ mice. In our study, we observed elevated mitochondrial ROS levels, reduced mitochondrial membrane potential, and significantly lower cellular ATP content. These findings demonstrate that NAD^+^ is vital for maintaining mitochondrial function in spermatocytes, highlighting its importance in spermatogenesis and male reproductive health.

Normal meiotic division in spermatocytes is essential for successful spermatogenesis (Caires, Broady, and McLean 2010), and the programmed repair of DSBs during prophase I of meiosis is essential for homologous recombination, ensuring proper chromosome segregation (Keeney, Giroux, and Kleckner 1997; Hunter 2015). Mitochondrial dysfunction has been increasingly recognized as a critical factor influencing meiotic recombination in spermatocytes (Durairajanayagam et al. 2021; Guo et al. 2023). Disruptions in mitochondrial dynamics, such as those caused by the loss of mitofusins, have been linked to defects in spermatocyte differentiation and meiotic progression, resulting in an accumulation of spermatocytes at early stages of meiosis (Varuzhanyan et al. 2019). Moreover, mitochondrial dysfunction has been associated with karyosome defects and delayed synaptonemal complex disassembly in oocytes, further underscoring the importance of mitochondrial health for chromatin organization and meiotic recombination (Nieken et al. 2023). Therefore, we also investigate whether *Qprt* knockout affects meiosis in spermatocytes, and further evaluated abnormalities in DSBs repair by examining the homologous recombination protein Rad51 and the crossover marker MLH1. Our findings confirmed that *Qprt* knockout disrupts meiotic prophase I progression and impairs DSBs repair. Additionally, our study revealed that *Qprt* knockout leads to abnormalities in meiotic sex chromosome inactivation. Pachytene spermatocytes with unsynapsed chromosomes or defective meiotic sex chromosome inactivation are typically cleared through apoptosis (Vernet et al. 2016). Previous research has shown that failure of meiotic sex chromosome inactivation during meiosis I is associated with germ cell developmental arrest and apoptosis (Fernandez‐Capetillo et al. 2003; Royo et al. 2010; Ichijima et al. 2011). Collectively, these findings suggest interplay between NAD^+^ metabolism and mitochondrial function appears to be critical for ensuring the fidelity of meiotic processes, and disruptions in this pathway may have profound implications for male reproductive health. Although this study found that *Qprt* is primarily expressed in spermatocytes within the testes, the potential regulatory roles of other tissues cannot be entirely excluded. For example, *Qprt* is expressed in the brain (Ishidoh et al. 2010), where it may influence neuroendocrine signaling pathways, and in the liver (Wang et al. 2017), where it supports NAD^+^ homeostasis critical for metabolic processes. Further investigation is needed to determine whether these tissues contribute to the regulation of testicular function.

In the present study, NR supplementation in *Qprt* ^*−/−*^ mice elevated NAD^+^ levels and improved spermatogenic outcomes, including testicular weight, sperm count, and seminiferous tubule morphology. NR also ameliorated meiotic defects, restoring progression and reducing DNA repair abnormalities during meiosis, particularly in chromosomal double‐strand break repair and crossover formation. These results highlight the therapeutic potential of NAD^+^ precursors in restoring spermatogenesis disrupted by NAD^+^ deficiencies. Given the role of NAD^+^ in metabolic processes and DNA repair, NAD^+^ boosting drugs such as NR and NMN may be beneficial in preserving fertility during aging, where NAD^+^ levels naturally decline, or in models with genetic defects affecting NAD^+^ metabolism in testis.

In conclusion, this study provides in vivo evidence that disruption of the NAD^+^ de novo synthesis pathway results in reduced NAD^+^ levels in both the testes and spermatocytes, leading to mitochondrial dysfunction and, ultimately, accelerated testicular aging. These findings deepen our understanding of the mechanisms underlying male infertility, particularly the critical role of NAD^+^ in maintaining mitochondrial health and genomic integrity during spermatogenesis. Furthermore, our results highlight the potential therapeutic benefits of supplementing NAD^+^ precursors as a strategy to mitigate testicular aging and address spermatogenic defects. Future research should focus on exploring these supplementation strategies to develop effective interventions aimed at improving male reproductive health.

## 4 Materials and Methods

### 4.1 Animals Feeding and Treatments

The *Qprt* knockout mice were created using the CRISPR/Cas9 genome editing technique. Genomic DNA was isolated from the tails of the mice using a DNA extraction kit (Vazyme). The extracted DNA was then subjected to Sanger sequencing to generate homozygous knockout mice. For NR supplementation treatment, four‐week‐old *Qprt* ^−/−^ mice were randomly allocated into two groups, one receiving standard feed, and the other supplemented with nicotinamide riboside (NR, Shanghai Biochempartner Co. Ltd) at a dosage of 400 mg/kg/day for a duration of 5–8 months. Control C57/BL6 mice were obtained from Beijing Vital River Experimental Animals Centre. All mice were kept in an SPF‐grade facility, under a 12 h light–dark cycle, at a controlled temperature of 20°C–25°C, with unrestricted access to food and water.

### 4.2 Histological Analysis

After deparaffinization, the testicular sections were rehydrated through a graded ethanol series. The sections were then treated with HD constant staining pretreatment solution for 1 min. Subsequently, the sections were processed sequentially with Hematoxylin solution, Hematoxylin Differentiation solution, and Hematoxylin Bluing solution. The sections were placed in 95% ethanol for 1 min, followed by Eosin dye for 15 s. Afterward, the sections were dehydrated sequentially with absolute ethanol, n‐butanol, and xylene, and then sealed with neutral gum. The sections were observed under a microscope (Nikon, DS‐Qi2, Japan), and the diameters of the seminiferous tubules were measured using NIS‐Elements BR software.

### 4.3 Testis Collection and Pachytene Spermatocytes Isolation

Following cervical dislocation euthanasia, testis was collected from the mice. The testis was fixed in 4% PFA solution or snap‐frozen in liquid nitrogen and stored at −80°C. After removing the tunica albuginea from the testis, the seminiferous tubules were digested enzymatically. The cell suspension was then filtered and washed. Pachytene Spermatocytes were subsequently isolated using a discontinuous density gradient of bovine serum albumin (BSA) as described previously (Da Ros et al. 2019).

### 4.4 Determination of NAD ^+^ Level in the Testis and Pachytene Spermatocytes

NAD^+^ levels were quantified using the NAD/NADH Assay Kit (Abcam) according to the manufacturer's protocol. Testes and pachytene spermatocytes were homogenized in lysis buffer, followed by centrifugation to collect the supernatant. Both the samples and the NAD^+^ Extraction buffer were pre‐heated to 37°C for 10 min. The NADH Extraction and NADH Reaction Mixture were then added to the tissue supernatant and incubated at room temperature for 2 h. Finally, fluorescence intensity was measured using a microplate reader (Molecular Devices, SpectraMax i3x + MinMax, USA), with excitation and emission wavelengths set at 540 and 590 nm, respectively.

### 4.5 Sperm Count and Morphology Analysis

The epididymal tissue in a 1 mL PBS eppendorf tube was minced using clean ophthalmic scissors and incubated at 37°C for 30 min to allow for sperm release. The sperm suspension was then collected, and 10 μL of the suspension was placed on a hemocytometer using a micropipette. After covering with a coverslip, the number of spermatozoa was counted under a standard light microscope (Nikon, DS‐Qi2, Japan). For sperm morphology observation, the sperm suspension was spread evenly onto a glass slide and air‐dried. The cells were then fixed with 95% ethanol, followed by staining with hematoxylin and eosin. The stained slides were observed under a microscope (Nikon, DS‐Qi2, Japan).

### 4.6 The Serum Testosterone Levels Assessments

The blood samples were allowed to clot for overnight at 4°C before centrifugation. The supernatant (serum) was carefully collected and stored at the appropriate temperature until further analysis. The serum testosterone levels in both groups were measured using a QuicKey Pro Mouse T (Testosterone) ELISA Kit (Elabscience).

### 4.7 ROS and Mitochondrial Membrane Potential Detection by Flow Cytometry

The pachytene spermatocytes were extracted from testes and incubated with MitoSOX Red Mitochondrial Superoxide Indicator (Invitrogen) for ROS detection or mitochondrial membrane potential assay kit (JC‐1) (Beyotime) for mitochondrial membrane potential detection at 37°C for 30 min. After washing three times with pre‐warmed PBS buffer, the single‐cell suspension was analyzed for fluorescence intensity using flow cytometry, respectively.

### 4.8 ATP Content Assessments

The isolated pachytene spermatocytes were washed three times with PBS, the cells were pelleted by centrifugation. The ATP content in both groups was measured using an ATP assay kit (Beyotime). Briefly, 400 μL of ATP lysis buffer was added to the cell pellets obtained from each testis, and the mixture was homogenized by pipetting. The samples were then centrifuged at 12,000 *g* for 5 min at 4°C, and the supernatant was collected for subsequent experiments. A standard solution was prepared for constructing the standard curve. The ATP detection working solution was prepared at a 1:9 ratio. Then, 100 μL of working solution and 20 μL of either sample or standard solution were added to an opaque black 96‐well plate, mixed thoroughly using a pipette, and incubated for 10 min at room temperature. Measure the absorbance using the luminometer function of a microplate reader (Molecular Devices, SpectraMax i3x + MinMax, USA), and then calculate the ATP content according to the manufacturer's instructions.

### 4.9 Spermatocyte Chromosome Spreads and Immunofluorescence Staining

The Spermatocyte nuclear surface spreading was prepared as previously detailed (Fan et al. 2021; Zhu et al. 2022). For immunofluorescence staining, after drying the slides, block the cells on the slides with 1% BSA and 0.1% Triton X‐100 in PBS. Subsequently, perform primary antibody staining related to DSB repair (SYCP1, SYCP3,γH2AX, Rad51 and MLH1). After incubating overnight at 37°C, bind the corresponding fluorescent secondary antibody (Alexa Fluor 555 and 488). Observe and quantify the results using immunofluorescence microscopy (Nikon, DS‐Qi2, Japan).

### 4.10 Immunofluorescence Staining on Testicular Sections

After deparaffinization of the testis sections, rehydrate them through a graded ethanol series. Then, perform antigen retrieval using a 0.1 mol/L citrate buffer solution at pH 6.0. The testicular sections were blocked with 3% BSA, 10% Donkey serum and 0.1% Triton X100 in PBS for 1 h at room temperature. Add the primary antibodies of QPRT (1:100, Proteintech) or VASA (1:100, Abcam) to the sections and incubate overnight at 4°C. After washing, add the corresponding fluorescent secondary antibodies (1:200, Alexa Fluor 555 and 488) and incubate at 37°C in the dark for 1 h. Following washing, mount the slides with an Antifade Mounting Medium with DAPI (Vector laboratories). The spermatocytes were observed and captured using immunofluorescence microscopy (Nikon, DS‐Qi2, Japan).

### 4.11 RT‐PCR and Western Blot Analysis

The method described previously was slightly modified and used to extract RNA from spermatocytes (Rio et al. 2010). The extracted RNA was reverse transcribed using the HIScript III RT SuperMix kit (Vazyme). Gene expression was then validated using the SYBR Green PCR Master Mix (Qiagen) on the QuantStudio 12 K Flex system (Applied Biosystems). The primer sequences are listed in Table S1. For the Western Blot (WB) analysis to assess protein expression levels in testis, proteins were extracted from the testis using a protein extraction kit (Sangon Biotech). The extracted proteins were then quantified with a protein assay kit (Bio‐Rad). After protein quantification, equal amounts of protein samples were prepared and loaded onto gels for separation via electrophoresis, and then transferred onto a PVDF membrane. The membrane was blocked with 5% skim milk for 1.5 h and incubated overnight with the primary antibody on a shaker. The next day, the membrane was washed six times with TBST, each wash lasting 10 min, and then incubated with the secondary antibody for 1 h at room temperature on a shaker. After three additional washes, the membrane was treated with a developing solution, and the target bands were visualized using a Bio‐Rad imaging system. The intensity of the bands was measured by ImageJ.

### 4.12 Statistical Analysis

Each experiment was repeated at least three times. Statistical comparisons were performed using Student's *t*‐test, one‐way ANOVA, and GraphPad Prism software. *p* < 0.05 was considered statistically significant.

## Author Contributions

Qingling Yang conceived the study. Qingling Yang, Yining Xu, and Huan Wang designed experiments. Yining Xu, Huan Wang, Hui Li, Chenlu Wei, Zhenye Zhu, Yanqing Zhao, Jiajia Zhu, Min Lei, Yingpu Sun and Qingling Yang performed experiments and data collection. Yining Xu and Huan Wang analyzed all data and prepared the figures. Yining Xu and Qingling Yang wrote the manuscript.

## Conflicts of Interest

The authors declare no conflicts of interest.

## Permission Statement

All copyrighted materials included in this manuscript (e.g., figures, tables, or text) have been used with permission from the respective copyright holders.

## Supporting information


**Figure S1.** The sperm morphology analysis in WT and *Qprt* ^*−/−*^ mice at 9 months of age.
**Figure S2**. The serum testosterone levels in WT and *Qprt* ^*−/−*^ mice at 9 months of age.
**Table S1**. List of primer sequences used for real‐time RT‐ PCR analysis.

## Data Availability

Data can be obtained from the corresponding author under reasonable request.
