# Supplementary material for: Nicotinamide Riboside Supplementation Alleviates Testicular Aging Induced by Disruption of Qprt‐Dependent NAD + De Novo Synthesis in Mice
Source: Aging Cell. 2025 Feb 4;24(6):e70004. doi: 10.1111/acel.70004 (PMC12151896; doi:10.1111/acel.70004)
Supplement: Supplementary file 1 — Figure S1. The sperm morphology analysis in WT and Qprt −/− mice at 9 months of age. Figure S2. The serum testosterone levels in WT and Qprt −/− mice at 9 months of age. Table S1. List of primer sequences used for real‐time RT‐ PCR analysis. [file ACEL-24-e70004-s001.docx]

**Supplementary Table1. List of primer sequences used for real-time RT- PCR analysis**

| Gene | Forward | Reverse |
| --- | --- | --- |
| Fth17 | CAGCAGGTCGACATTTTGAA | GGCTGAGCTTGTCAAAGAGG |
| Usp26 | AATGTAACGAAGGGAGAAGTG | AGGCTTTGCCTTCTTATCGAG |
| Atrx | GCTTGTGGACAGCAGGTCAAT | GTCACGGCTAATATCGTCACTC |
| Tktl1 | TCAAAGGGACTACCATTTGTT | AACAGGGGGCGAAGTCATACA |
| Rbmy | AACCGAAGTAACATATACTCA | ATCTGCTTTCTCCACGACCTC |
| Ubely | ATTGACTTTGAGAAGGATGAC | CAGACACACAAGGCCAACTAT |
| β-actin | GGCACCACACCTTCTACAATG | GTGGTGGTGAAGCTGTAGCC |

**
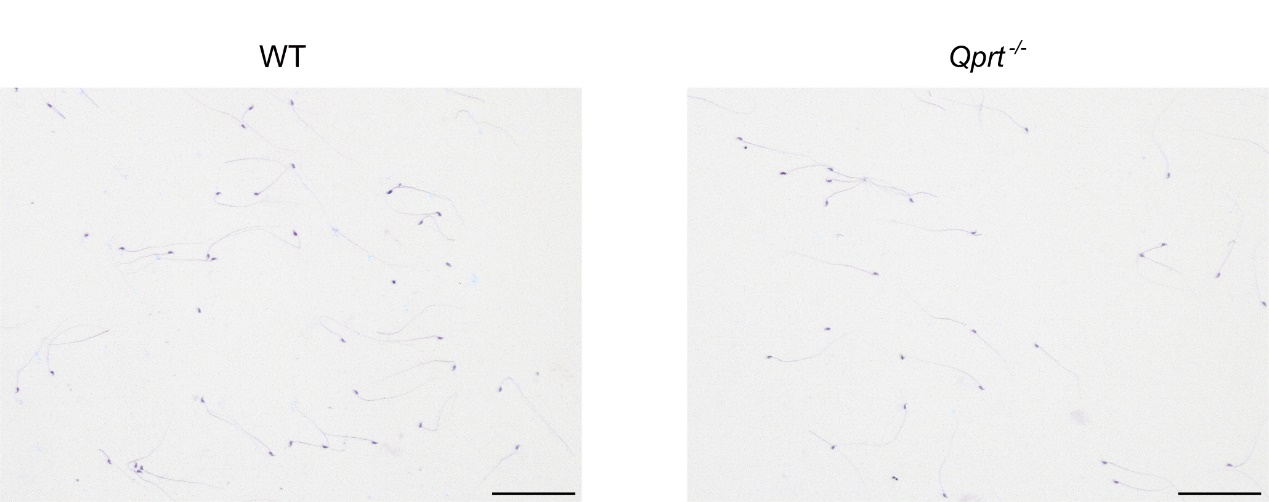
**

**Supplementary Figure 1. The sperm morphology analysis in WT and *Qprt^-/-^* mice at 9 months of age.**

Representative sperm morphology staining for WT and *Qprt*^-/-^ mice at 9 months of age. Scale bar = 100 μm.

**
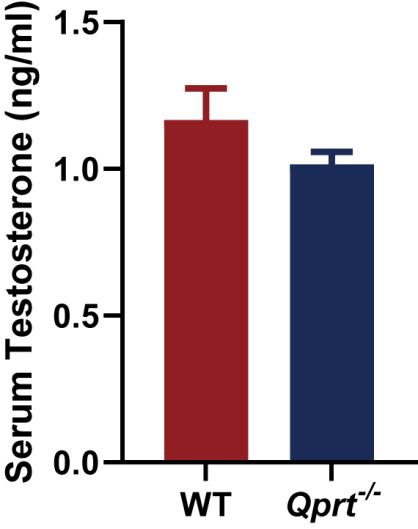
**

**Supplementary Figure 2.** **The serum testosterone levels in WT and *Qprt^-/-^* mice at 9 months of age.**

The serum testosterone levels in WT and *Qprt^-/-^* mice at 9 months of age (n = 5 mice for each group).
